# Vortex equilibria using least-squares methods

**DOI:** 10.1007/s00162-025-00746-0

**Published:** 2025-06-28

**Authors:** Samuel J. Harris, N. R McDonald

**Affiliations:** Department of Mathematics, University College London, Gower St, London, WC1E 6BT UK

**Keywords:** Vortex dynamics, Laplace’s equation, Rational approximation, AAA-least squares, Lightning Laplace solver

## Abstract

Numerical methods and results for computing rotating or stationary equilibria of vortex patches and sheets, some in the presence of point vortices, are presented. The methods are based on those recently developed by Trefethen and colleagues for solving Laplace’s equation in the complex plane by series and rational approximation. They share the common feature of finding the coefficients of the approximation by the fitting of boundary conditions using least-squares. Application of these methods to vortex patches requires their extension to the solution of Poisson’s and Laplace’s equation in two domains with matching conditions across the patch boundary. In the case of vortex sheets, the streamlines of the solution are computed along with the circulation density of the sheet. The use and accuracy of the methods is demonstrated by reproducing known results for equilibrium patches and vortex sheets, some having point vortices present. Several new numerical equilibrium solutions are also computed: a single straight sheet with two and four satellite point vortices respectively, and a three-sheeted structure, with the sheets emanating from a common point of rotation. New numerical solutions are also found for steady, doubly-connected vortex layers of uniform vorticity surrounding solid objects and such that the fluid velocity vanishes on the outer free boundary.

## Introduction

Persistent, coherent vortex structures arise in diverse physical systems such as, for example, the quasi-two dimensional flow of fluids in the Earth’s oceans and the atmospheres of the giant planets, or in strongly magnetized systems involving electron columns confined in Malmberg–Penning traps [1–3]. The longevity of these vortical structures suggests that they are equilibrium solutions of the governing equations. In the context of the two-dimensional Euler equations, the systematic search and study of equilibrium vortex solutions has a rich history. In its simplest, yet surprisingly rich, guise, this amounts to finding configurations of point vortices which are stationary in either rotating or translating frames (including stationary frames). There is a considerable body of literature on this topic which continues to receive recent attention e.g. [4–6].

The desingularization of point vortices into vortex sheets and patches represents a more complex form of vortical structure. Vortex sheets comprise of two-dimensional curves along which vorticity is continuously distributed. The search for vortex sheet equilibria is challenging since both the vorticity distribution (or, equivalently, the circulation density) along the sheet and the shape of the curve must be found. Beyond a single finite length sheet rotating about its centre e.g. [7, 8], most known exact solutions fall into the class of an ensemble of straight sheets forming rotating equilibria e.g. [9, 10]. Computationally, the natural approach to seeking sheet equilibria is to formulate and solve a singular integral equation (the Birkhoff-Rott equation–see [8]) in either a rotating or translating frame. Another approach is to approximate the sheet by a collection of point vortices of varying strength distributed along a curve of finite length [11].

Vortex patches have a finite, non-zero area of constant vorticity and, again, the main challenge lies in finding the shape of the equilibrium patch, and so, mathematically-speaking, essentially becomes a two-dimensional free boundary problem. Contour dynamics methods in which the velocity field is numerically computed through knowledge of the boundary shape have proved successful in finding families of both translating and rotating equilibria e.g. [12–15]. As is the case for vortex sheets, exact solutions for equilibrium vortex patches are similarly scarce: in addition to the classic Kirchhoff vortex, an elliptical vortex patch in steady rotation [8], analytic progress in finding equilibrium rotating solutions is possible only for limited classes e.g. using techniques based on conformal mapping and the Schwarz function of the patch boundary e.g. [16–18].

The purpose of this work is to demonstrate the use of recently-developed methods by Trefethen and colleagues for the numerical solution of the two-dimensional Laplace’s equation in finding rotating vortex equilibria. The emphasis here is on the tailoring, extension and application of these methods to vortex problems. In a series of papers [19–23] methods based on series expansion and rational approximation in the complex plane of the unknown function in conjunction with least-squares fitting of boundary data have proved considerably effective in the rapid computation of highly accurate solutions of Laplace’s equation. Such methods are referred to here as ‘least-squares methods’ since the procedure of satisfying boundary data via this procedure is common to them all.

One particular least-squares method employs the AAA algorithm (e.g. [19, 23]) which finds a rational approximation of a function in the complex plane. It is adept at solving Laplace’s equation in either interior or exterior domains, which may be multiply connected and have corners on its boundaries, and subject to either Dirichlet or Neumann boundary data. In a fluid dynamics context, the method gives both the real and imaginary part of the complex potential function, so giving both the velocity potential and the stream function. However, the solution of vortex patch problems requires some further development of the method. First, inside the patch the governing equation is Poisson’s equation with a constant right hand side equivalent to the constant vorticity $\omega$ of the patch, and, second, that both Poisson’s equation inside the patch and Laplace’s equation outside the patch (including the point at infinity) must be simultaneously solved for. Typically, the latter requires appropriate consideration of the velocity field across the patch boundary, so that the boundary conditions are of the continuity type. Solving Poisson’s equation is straightforward by introducing a particular solution and adjusting the boundary conditions appropriately; the choice $\omega (x^2+y^2)/4$ for the particular solution is made here. The solution to Laplace’s problem with the adjusted boundary conditions can then be sought using AAA-least squares (AAA-LS). A similar approach involving a different application was performed in [24] for an interior-only problem involving Poisson’s equation. Recently [25] have applied an adaption of AAA-LS to compute the field produced by permanent magnets, where the interface boundary condition on the interface requires the inclusion of a surface current density which is then used to solve the interior and exterior harmonic problems.

In the case of a singly connected patch, the second development of finding simultaneous solutions in both the interior and exterior domains is referred to here as the two-domain Laplace problem. The authors have previously described the extension of the AAA-LS method to two-domain Laplace problems with application to bio-electrostatics problems in [26], and one motivating reason for the present work is to highlight another application of this extension. Section 2 describes the rotating vortex patch equilibrium problem and Sect. 3 details the solution procedure using the two-domain AAA-LS method. Numerical tests are performed in Sect. 4 by comparing with the Kirchhoff elliptical vortex, and the results of Wu *et al.*’s [13] contour dynamics method are reproduced in Sect. 5. Rotating equilibria involving both patches and point vortices are computed in Sect. 6 and compared to that computed by [15].

On the vortex sheet problem, two classes of equilibria are computed using least-squares methods. The first of these is in Sect. 7.1 in which Trefethen’s [20] series solution of Laplace’s equation is used to derive solutions involving straight vortex sheets rotating in equilibrium with point vortices. These solutions, two of which have not been previously reported, are compared to exact solutions derived using techniques from [9]. The second class, in Sect. 7.2, is that of the multi-sheet rotating equilibria derived by [10]. In this case, the log-lightning method of [22] is used to reproduce the solutions of [10], and to find a new solution with an odd number (three) of sheets stemming from the centre of rotation.

Stationary equilibria comprising of doubly-connected, two-dimensional vortex layers surrounding a solid object, and such that the fluid velocity vanishes identically on the outer, free boundary, are computed using the doubly connected AAA-LS algorithm in Sect. 8. Example numerical solutions for the free boundary are given.

## Vortex patch model

**Fig. 1:**
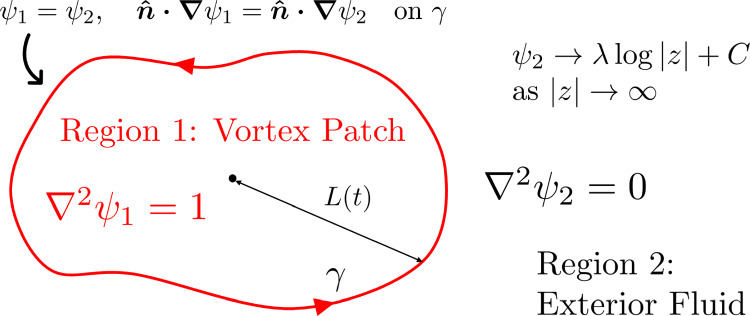
The singly connected vortex patch problem showing the non-dimensional governing equations for the stream functions $\psi _1$ and $\psi _2$, boundary conditions on the interface $\gamma$ of the two domains (regions 1 and 2) and the condition at infinity

Consider the singly connected vortex patch problem sketched in Fig. 1. The vortex patch has (conformal) radius *L* and positive (without loss of generality) constant vorticity $\omega$. Define the Jordan curve $\gamma$ as the boundary separating the interior vortex patch (region 1) and the exterior fluid (region 2). Both interior and exterior fluids are incompressible and inviscid, and the exterior flow is irrotational. The stream function $\psi$ and the velocity $\varvec{u}$ are continuous across the boundary $\gamma$; the vorticity jumps from zero to $\omega$. In the far-field, the vortex patch appears as a line vortex with circulation $\Gamma =\omega A$, where *A* is the area enclosed by $\gamma$. The scalings $\varvec{x}=L\varvec{x}^*$ and $\psi = (\omega L^2)\psi ^*$ are introduced, where starred variables are dimensionless. Dropping stars immediately, the non-dimensional system is thus

1 $$\begin{aligned} \nabla ^2\psi _1=1\;\;\;\text { in 1,} \end{aligned}$$

2 $$\begin{aligned} \nabla ^2\psi _2=0\;\;\;\text { in 2,} \end{aligned}$$

3 $$\begin{aligned} \psi _1=\psi _2\;\;\;\text { on }\gamma , \end{aligned}$$

4 $$\begin{aligned} \varvec{\hat{n}\cdot \nabla }\psi _1=\varvec{\hat{n}\cdot \nabla }\psi _2\;\;\;\text { on }\gamma , \end{aligned}$$

5 $$\begin{aligned} \psi _2\rightarrow \lambda \log |z| + C \;\;\;\text {as }r\rightarrow \infty , \end{aligned}$$

where $\lambda = A/(2\pi L^2)$ and the constant $C=A\log |L|/(2\pi L^2)$; the value $L=1$ is taken throughout this work unless stated otherwise and thus the constant *C* can be ignored. To find the velocity vector, note that $\varvec{u}=\varvec{\hat{z}}\times \varvec{\nabla }\psi =-\psi _y\varvec{\hat{x}}+\psi _x\varvec{\hat{y}}$. Using complex notation where $\nabla =\partial _x +i\partial _y$, the velocity is $u+i v = i\nabla \psi =i(\psi _x+i\psi _y)=-\psi _y+i\psi _x$.

## Two-domain AAA-LS algorithm

Numerical solutions to the system (1) − (5) are found using the AAA-LS algorithm which has been in continuous development by Trefethen, Costa, Gopal and their colleagues for over five years [27]. This method computes a rational approximation of a harmonic function in a bounded interior (or unbounded exterior) domain, consisting of a polynomial plus a finite sum of simple poles. The method uses both the AAA algorithm, which finds suitable poles (in the unphysical region) for given boundary data, plus a least-squares fit to this data. In this work, two extensions to the method must be made. First, the substitution $\psi _1 = \widetilde{\psi _1} + |z-z_c|^2/4$ is made transforming the interior Poisson equation (1) into Laplace’s equation for $\widetilde{\psi _1}$. Note that $z_c$ is the centre of the vortex patch, though it is assumed that $z_c=0$ throughout this work. The exterior stream function $\psi _2$ is also modified as $\psi _2 = \widetilde{\psi _2} + \lambda \log {|z-z_c|}$ so that $\widetilde{\psi _2}$ decays in the far-field. The functions $\widetilde{\psi _1}, \widetilde{\psi _2}$ can be written as $\widetilde{\psi _i}=\text {Re}[F_i(z)]$ where $F_i(z)$ is analytic in the respective domain of $\widetilde{\psi _i}$ [21, 23]. A rational approximation for each *F* can then be written as

6 $$\begin{aligned} \psi _1= &   \frac{|z|^2}{4}+\text {Re}[F_1(z)]= \frac{|z|^2}{4}+\text {Re}\bigg (\sum _{k=0}^{N_1} a_k (z-z_c)^k + \sum _{k=1}^{M_1}\frac{b_k}{z-p_k}\bigg ), \end{aligned}$$

7 $$\begin{aligned} \psi _2= &   \lambda \log |z| + \text {Re}[F_2(z)] = \lambda \log |z| + \text {Re}\bigg (\sum _{k=1}^{N_2} c_k (z-z_c)^{-k}+\sum _{k=1}^{M_2}\frac{d_k}{z-q_k}\bigg ), \end{aligned}$$

where $N_1$ and $N_2$ are series truncations; the values $N_1=N_2=20$ are typically used in this work.

Second, the AAA-LS algorithm is extended to solve for $\psi _1$ and $\psi _2$ simultaneously in this two-domain, mixed boundary value problem. This is achieved by a rewriting of the two boundary conditions (3) and (4) as follows: substituting (6) and (7) into the Dirichlet condition (3) and rearranging gives

8 $$\begin{aligned} \text {Re}[F_1(z)]-\text {Re}[F_2(z)] = \lambda \log {|z|}-\frac{|z|^2}{4}=H_1(z). \end{aligned}$$

For the Neumann boundary condition (4), note that $\varvec{\hat{n}\cdot \nabla }\psi = \text {Re}[n\overline{\nabla }\psi ]$, where $n=n_x+in_y$ and $\nabla$ are complex representations of the corresponding vectors. Note also that $\overline{\nabla }=2\partial _z$ and that $|z|^2=z\overline{z}$. Further, equation (5) from [20] gives that $\overline{\nabla }[\text {Re}[F(z)]] = F'(z)$. Therefore, the condition (4) becomes

9 $$\begin{aligned} \text {Re}[nF_1'(z)]-\text {Re}[nF_2'(z)] = \lambda \text {Re}[nz^{-1}]-\frac{1}{2}\text {Re}[n\overline{z}]=H_2(z). \end{aligned}$$

These conditions can now be written as the product $Ac=H$ where *A* is a matrix of suitable basis vectors, $c = [a_k; b_k; -c_k; -d_k]$ is the vector of unknown coefficients and $H=[H_1(z); H_2(z)]$ is a vector of given functions. An Arnoldi orthogonalisation can be implemented in the construction of the Vandermonde matrix *A* as in [28], though this was not performed in this work as solutions were sufficiently stable. The unknown *c* can then be found with a standard least-squares fitting procedure using the backslash operator $c=H\backslash A$.

Simple poles are found with the AAA algorithm using both the functions $\text {Re}[H_1]$ and $\text {Re}[H_2]$. The union of these poles are then taken and separated into the exterior $p_k$ and interior $q_k$ poles as used in (6) and (7). To avoid numerical errors known as Froissart doublets (see e.g. [19]), poles with a sufficiently small residue below $\mathcal {O}(10^{-8})$ and those sufficiently close to the vortex patch boundary (of $\mathcal {O}(10^{-2})$) are manually removed.

To find the velocity vector in each region, recall that $u+iv=i\nabla \psi$ and that $\nabla [\text {Re}[F(z)]] = \overline{F'(z)}$. Therefore

10 $$\begin{aligned} u_1+iv_1&= i\bigg ( \frac{z}{2}+\overline{\sum _{k=0}^{N_1} ka_k (z-z_c)^{k-1} - \sum _{k=1}^{M_1}\frac{b_k}{(z-p_k)^2}}\bigg ), \end{aligned}$$

11 $$\begin{aligned} u_2+iv_2&= i\bigg (\frac{\lambda }{\overline{z}} - \overline{\sum _{k=1}^{N_2} kc_k (z-z_c)^{-(k+1)}-\sum _{k=1}^{M_2}\frac{d_k}{(z-q_k)^2}}\bigg ). \end{aligned}$$

The accuracy and robustness of the AAA-LS algorithm can then be tested by comparing its outputs with known solutions. This is done in the following three sections.

## Kirchhoff vortex

An exact equilibrium solution of the system (1) − (5) is known - see chapter 9 of [8] and the references therein. This is the Kirchhoff vortex - an elliptical vortex patch of constant vorticity $\omega$ with semi axes *a* and *b* and, in a rotating frame, a fixed angle $\varphi$ between its major axis and the real *z* axis. Note, without loss of generality, the choice $\varphi =0$ can be made but a non-zero choice is made here in order to provide a more stringent test of the method. The conformal map from the exterior of the unit disk to the exterior of this ellipse is

12 $$\begin{aligned} z = e^{i\varphi }\Big (\alpha \zeta +\frac{\beta }{\zeta }\Big ), \;\;\;\; \alpha = \frac{a+b}{2},\;\; \beta = \frac{a-b}{2}. \end{aligned}$$

By definition, the conformal radius is $\alpha =L$ and so define the new dimensionless quantity $B=\beta /L$, and use $\omega ^{-1}$ as the timescale. On the boundary of the ellipse, the Schwarz function $\overline{z}=\Phi (z)$ can be written as

13 $$\begin{aligned} \overline{z} = \bigg (Be^{-2i\varphi }z\bigg ) + \bigg (\frac{2(1-B^2)}{z+\sqrt{z^2-4Be^{2i\varphi }}}\bigg ) = F(z)+G(z). \end{aligned}$$

By equation (9.2.11) from [8], the velocity field is defined by

14 $$\begin{aligned} u-iv = {\left\{ \begin{array}{ll} -\frac{i}{2}(\overline{z}-F(z)), \;\;\; z\in 1, \\ -\frac{i}{2}G(z), \;\;\; z\in 2, \end{array}\right. } \end{aligned}$$

and therefore

15 $$\begin{aligned} u-iv = {\left\{ \begin{array}{ll} -\frac{i}{2}(\overline{z}-Be^{-2i\varphi }z), \;\;\; z\in 1, \\ -i(1-B^2)/(z+\sqrt{z^2-4Be^{2i\varphi }}), \;\;\; z\in 2. \end{array}\right. } \end{aligned}$$

**Fig. 2:**
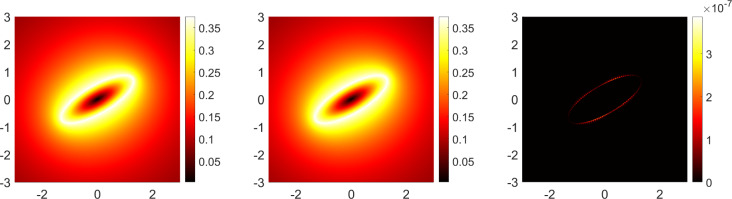
Comparison of the AAA-LS and exact solutions for a Kirchhoff vortex with semi axes $a=1.5$ and $b=0.5$ and at an angle $\varphi =\pi /6$ to the real axis. The relative error (right column) between the AAA-LS (left column) and exact (centre column) solutions is found for the velocity magnitude

The AAA-LS method is used to compute an example Kirchhoff vortex with $\varphi =\pi /6$ and semi axes $a=1.5$ and $b=0.5$. The AAA-LS solutions are shown in the left column of Fig. 2, with the exact solutions given in the centre column. To test the accuracy of the AAA-LS method in comparison to the exact solutions, the following relative error (RE) of known quantities is found

16 $$\begin{aligned} RE = \bigg |\frac{\Phi _A - \Phi _E}{\Phi _E}\bigg | \end{aligned}$$

where $\Phi _A$ is the quantity calculated by the AAA-LS method and $\Phi _E$ the exact solution. For cases where $\Phi _E\approx 0$ (in particular the criteria $\Phi _E=\mathcal {O}(10^{-4})$ is used in this work), the absolute error (AE) is instead used

17 $$\begin{aligned} AE = \big |\Phi _A - \Phi _E\big |. \end{aligned}$$

For the Kirchhoff vortex, the magnitude of the velocity is the compared quantity, thus $\Phi = |u+iv|$ where $\Phi _E$ is found by taking the magnitude of the exact velocity $u-iv$ in equation (15). For each point in the $500\times 500$ grid of each subplot of Fig. 2, the relative error of the compared quantity is calculated and plotted in the right column. It is found that there is an $\mathcal {O}(10^{-7})$ relative error which is comparable to the error for solving Laplace’s equation with Dirichlet boundary conditions in one domain [21, 23].

**Fig. 3:**
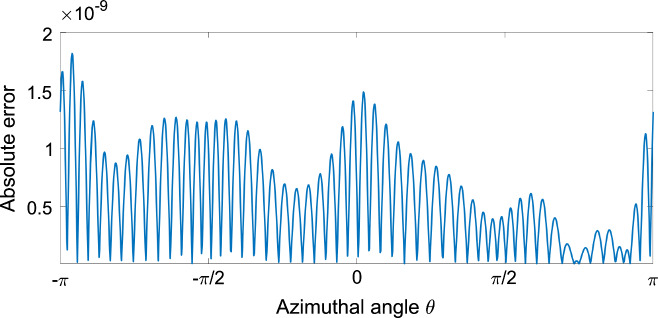
Absolute error of the normal velocity on the vortex patch boundary of the AAA-LS solution in the equilibrium frame

The Kirchhoff ellipse rotates steadily with angular velocity $\Omega$ given by [8]

18 $$\begin{aligned} \Omega = \frac{1-B^2}{4}, \end{aligned}$$

which has been non-dimensionalised as in Sect. 2. Subtracting the associated stream function $\psi = \Omega r^2/2$ and velocity $u+iv=i\Omega z$ from the corresponding global quantities then gives a vortex patch with zero normal velocity on its boundary i.e. $\varvec{\hat{n}\cdot v}=v_n=0$. That is, its boundary is a streamline as required for equilibrium. The error in this normal velocity is obtained using $\Phi = v_n$ in equation (17); note that the absolute error is used here since $\Phi _E=0$. Figure 3 shows this absolute error at boundary points with $\textrm{arg}(z)=\theta$ of the ellipse in Fig. 2; the AAA-LS solution is accurate to $\mathcal {O}(10^{-9})$.

## Wu, Overman and Zabusky equilibria

Wu, Overman and Zabusky [13] numerically compute $m-$fold symmetric equilibrium solutions (‘V-states’) to the vortex patch system (1) − (5). The aim of this section is to combine their algorithm (referred to as ‘WOZ’ hereafter) with the AAA-LS method developed in Sect. 3, and reproduce the results from [13] which uses contour dynamics to compute the velocity field.

Since an $m-$fold symmetric equilibrium necessarily retains its symmetry throughout the iterative process, only a 1/2*m* segment of the boundary need be considered. The vortex patch boundary $\gamma$ consists of *N* equispaced boundary points, where *Z* is the list of these points $z=x+iy$. Then $Z_s$ is the list of the $M+1<N$ points which make up the desired 1/2*m* segment of the boundary. At each iteration *n*, the new radial distance $R(\theta )^{(n+1)}$ is found for each boundary point with $\arg (z) = \theta$ and the boundary updated accordingly. The end points $z_1$ and $z_{M+1}$ are held fixed with $R_1^{(n)}=R_1^{(1)}=R_a$ and $R_{M+1}^{(n)}=R_b$ for all iterations *n* where $R_a$ and $R_b$ are some constants. A summary of the WOZ numerical procedure is given in appendix A.

Consider the $m-$fold symmetric rotating V-states investigated in Sect. 6.1 of [13]. For a given starting value of $R_a$, the initial state of the vortex patch is defined by

19 $$\begin{aligned} R(\theta ) = R_a + \frac{m^2}{\pi ^2}(1-R_a)\Big (\theta +\frac{\pi }{2}\Big )\bigg (\frac{2\pi }{m}-\theta -\frac{\pi }{2}\bigg ), \end{aligned}$$

for the 1/2*m* segment of the vortex patch boundary $-\pi /2\le \theta \le \pi /m - \pi /2$; note that this fixes $R_b=1$. The results from the AAA-LS + WOZ algorithm are compared with the results in [13] for the symmetries $3\le m\le 6$ with a selection of $M=120$ points taken on each 1/2*m* segment, following the choice in [13]. The iteration process in each case continues until the tolerance (A5) is reached or until $n_{max}=500$ iterations are computed. Note that the AAA-LS solutions are not scaled with respect to $R_a$ to allow for easier direct comparison with the WOZ solutions.

**Fig. 4:**
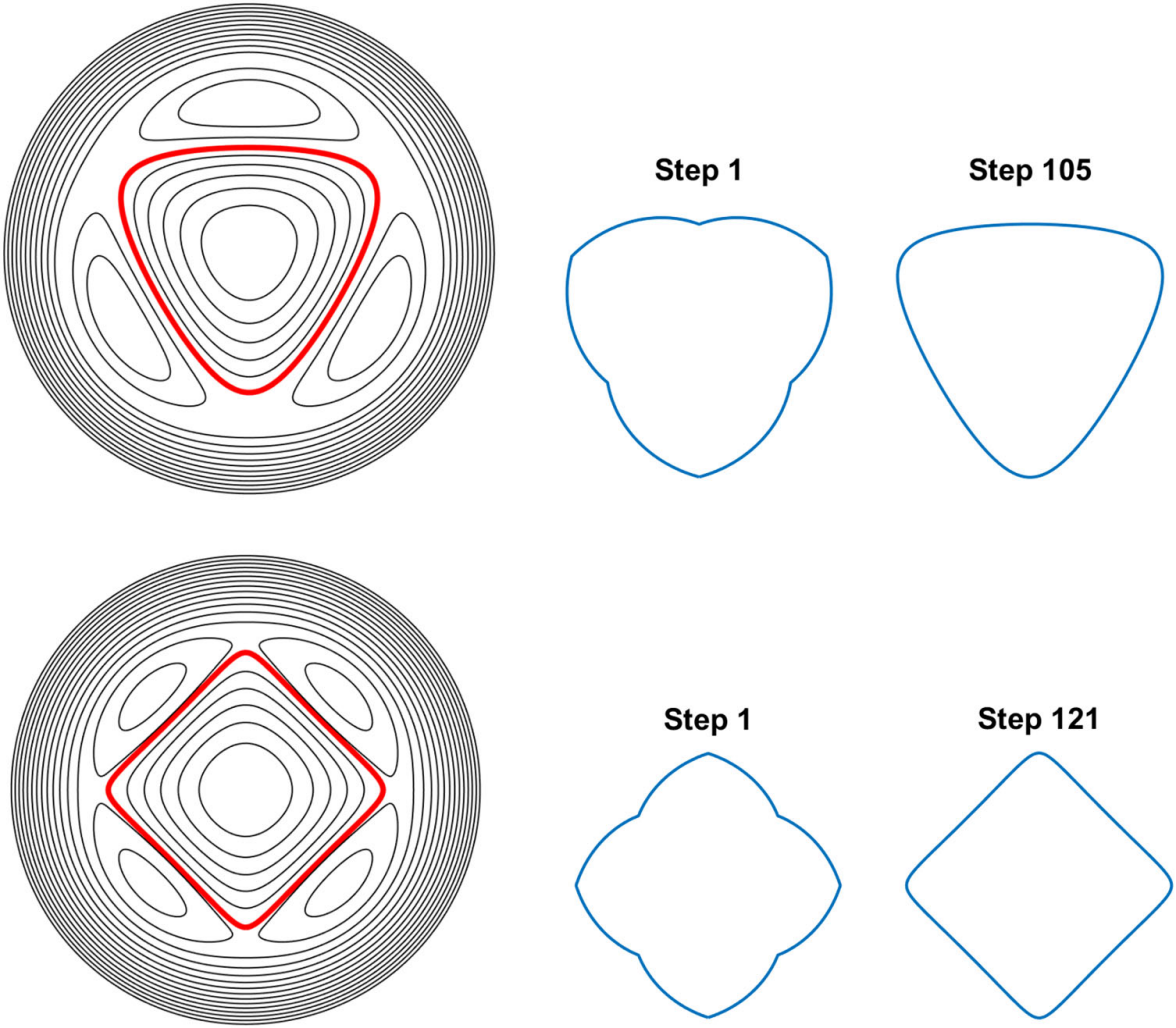
Streamline plots of the equilibrium solutions of the AAA-LS + WOZ algorithm on $m-$fold rotating V-states (left, the boundary of the patch is shown in red), with separate plots of the initial (centre) and final (right) shapes of the vortex patch. Top: three-fold shape, $R_a=1.39256$, 105 iterations. Bottom: four-fold shape, $R_a=1.34127$, 121 iterations (colour figure online)

Figure 4 displays streamline plots of the equilibrium solutions for the three- (top) and four-fold (bottom) rotating V-shapes (left, with the boundary of the vortex patch highlighted in red), where the initial (centre) and final steady state (right) shapes are also plotted separately. The choice of $R_a$ for each shape is given in the figure caption as is the number of iterations computed. There is a qualitative agreement between the results in Fig. 4 and those displayed in e.g. Fig. 7 of [13]. For a quantitative comparison, the values of the angular velocity, area and perimeter of the final equilibria are compared with the associated values listed in table III of [13]. The relative error (16) is calculated for each quantity and the results are as follows: the angular velocity agrees to $\mathcal {O}(10^{-4})$; the area agrees to $\mathcal {O}(10^{-5})$ and; the perimeter agrees to $\mathcal {O}(10^{-6})$. There is thus qualitative and quantitative agreement between the solutions of [13] and the AAA-LS + WOZ algorithm.

Furthermore, the typical runtime of one iteration of the AAA-LS + WOZ algorithm on a standard laptop is around 0.65 seconds. This value is closer to a step per second for higher fold symmetries, which is expected as these contain more boundary points for the same value $M=120$.

## Point vortex - vortex patch equilibria

A natural extension to the problem set up in Sect. 2 is to consider an $m-$fold symmetric vortex patch surrounded by *m* symmetrically positioned point vortices outside of the patch. This problem was studied in [15, 29] and labelled as the ‘$m+1$ point vortex − vortex patch equilibrium’. The system of governing equations and boundary conditions (1) − (4) are unchanged, however the far-field condition (5) is now

20 $$\begin{aligned} \psi _2\rightarrow \lambda \log |z| + \sum _{j=1}^m \lambda _s\log |z-z_j| + D\;\;\;\text {as }r\rightarrow \infty , \end{aligned}$$

where $z_j = (b/L)\exp {(2\pi ij/m)}$ is the (dimensionless) position of the $j^{\text {th}}$ point vortex and *b* is the radial distance of the point vortices from the origin. Each point vortex has the same circulation $\Gamma _s$ therefore $\lambda _s=\Gamma _s/2\pi \omega L^2$ and $D=(A+m\Gamma _s/\omega )\log |L|/(2\pi L^2)$ following the non-dimensionalisation in Sect. 2. The characteristic length *L* is now chosen to be the radial distance *b* and so $b=1$ is normalised throughout this work, once again giving $D=0$.

By linearity, the point vortices contribution can simply be added to both interior (6) and exterior (7) stream functions, giving

21 $$\begin{aligned} \psi = {\left\{ \begin{array}{ll} \psi _1 = \frac{|z|^2}{4}+ \sum _{j=1}^m \lambda _s\log |z-z_j|\\ \;\;\;\;+\;\text {Re}\bigg (\sum _{k=0}^{N_1} a_k (z-z_c)^k + \sum _{k=1}^{M_1}\frac{b_k}{z-p_k}\bigg ), \;\;\; z\in 1, \\ \psi _2 = \lambda \log |z| + \sum _{j=1}^m \lambda _s\log |z-z_j|\\ \;\;\;\;+\; \text {Re}\bigg (\sum _{k=1}^{N_2} c_k (z-z_c)^{-k}+\sum _{k=1}^{M_2}\frac{d_k}{z-q_k}\bigg ), \;\;\; z\in 2, \end{array}\right. } \end{aligned}$$

and using $u+iv=i\nabla \psi$, the velocity vector (in complex notation) is

22 $$\begin{aligned} u+iv = {\left\{ \begin{array}{ll} u_1+iv_1 = i\bigg ( \frac{z}{2}+ \sum _{j=1}^m \lambda _s/(\overline{z-z_j})\\ \;\;\;\;+\;\overline{\sum _{k=0}^{N_1} ka_k (z-z_c)^{k-1} - \sum _{k=1}^{M_1}\frac{b_k}{(z-p_k)^2}}\bigg ), \;\;\; z\in 1, \\ u_2+iv_2 = i\bigg (\frac{\lambda }{\overline{z}} + \sum _{j=1}^m \lambda _s/(\overline{z-z_j})\\ \;\;\;\; -\; \overline{\sum _{k=1}^{N_2} kc_k (z-z_c)^{-(k+1)}-\sum _{k=1}^{M_2}\frac{d_k}{(z-q_k)^2}}\bigg ), \;\;\; z\in 2. \end{array}\right. } \end{aligned}$$

Three of the four parameters $[R_a, R_b, b$ and $\gamma _s]$ must be prescribed to uniquely determine the solution. By freeing the parameter $R_b$, the WOZ algorithm detailed in appendix A can once again be used to numerically iterate towards equilibrium solutions. To do this, the algorithm is now implemented on a 1/*m* segment of the vortex patch (rather than a 1/2*m* segment as before) made up of $2M+1$ points such that $R_b=R_{2M+1}=R_1=R_a$. The expression (A1) for velocity $\Omega ^{(n)}$ at step 2 of the WOZ algorithm can no longer be used since $R_b=R_a$. Instead, the condition that the point vortex is fixed in the rotating frame is used giving the alternative expression for the angular velocity

23 $$\begin{aligned} \Omega ^{(n)} = |u(\Gamma _s,b,z_k^{(n)})|/b, \end{aligned}$$

where $z_k^{(n)}=x_k^{(n)}+iy_k^{(n)}$ is the $k^{\text {th}}$ boundary point at the $n^{\text {th}}$ iteration and |*u*| is the speed at a point vortex; by symmetry, each point vortex experiences the same speed. A given point vortex does not interact with itself, thus the corresponding singular term at $z=z_j$ is omitted in the velocity equation (22) at that point vortex.

**Fig. 5:**
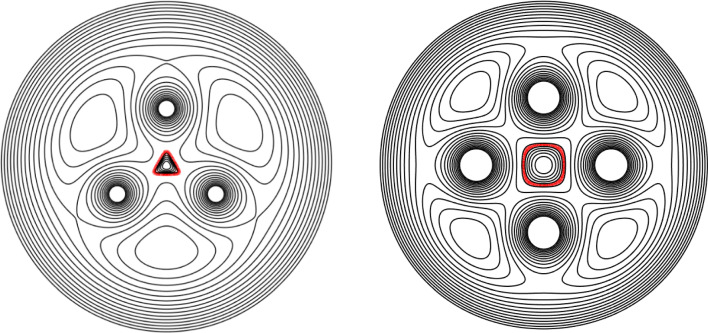
Streamline plots of the equilibrium solutions of the AAA-LS + modified WOZ algorithm for the point vortex-vortex patch problem, where the boundary of the vortex patch is shown in red in each Fig. Left: three-fold problem with same signed circulation of the patch and point vortices, where $|\Gamma _s|=\pi /2$ and $R_a=0.25$. Equilibrium computed in 55 iterations. Right: four-fold problem with opposite signed circulation of the patch and point vortices, where $|\Gamma _s|=2\pi$ and $R_a=0.3$. Equilibrium computed in 9 iterations (colour figure online)

Figure 5 shows streamline plots of two equilibrium solutions having three and four-fold symmetries, where the initial vortex patch shape is a circle of radius $R_a$ in each case. In the three-fold example (left), the point vortices have the same signed circulation as the vortex patch, with $|\Gamma _s|=\pi /2$, $R_a=0.25$ and the equilibrium is reached after 55 iterations. In the four-fold example (right), the point vortices have oppositely signed circulation to the vortex patch; the equilibrium is reached in 9 iterations, with $|\Gamma _s|=2\pi$ and $R_a=0.3$.

## Vortex sheet equilibria

Two classes of equilibria involving vortex sheets are considered in this section using numerical methods for solving Laplace’s equation by series approximated by applying least-squares to boundary data along the sheet. While some new examples are presented, the primary aim is to demonstrate how to use the numerical method in this context, and to promote it as a way to find and investigate new families of equilibria.

### Sheets and point vortices

Trefethen’s least-squares based algorithm for the numerical solution of Laplace’s equation by series [20] is used to compute rotating equilibria involving a single, straight vortex sheet of finite length in combination with point vortices. New numerical examples are presented and checked using the exact, complex-valued rational function method developed by [9].

Section 4 of [20] gives a numerical procedure for computing the Green’s function outside a slit $c+r[-1,1]$ where *c* and *r* are complex numbers specifying the slit. In this work, *c* and *r* are assumed to be real so the slit is aligned with the real axis and of length 2*r*. The slit coincides with the vortex sheet. The conformal map from the exterior of the unit disk in the *w*-plane to the exterior of the slit in the *z*-plane is

24 $$\begin{aligned} z=c+r(w+w^{-1})/2, \end{aligned}$$

with inverse

25 $$\begin{aligned} w=z_c+\sqrt{z_c^2-1/2},\quad z_c=(z-c)/r, \end{aligned}$$

with care needed to choose the correct branch of the square root [20].

Suppose a point vortex of strength $\Gamma$ is located at $z_{pv}\notin c+r[-1,1]$. Solutions are sought in which the combined point vortex and sheet form a rotating equilibrium, so that when there exists a background uniformly rotating flow with stream function $-\Omega |z|^2/2$, the sheet and point vortex become stationary. Here $\Omega$ is the angular velocity of the background flow and is to be found, along with the circulation density $\rho (x)$ along the sheet.

In a straightforward adaption of [20], an approximate form for the solution of the stream function exterior to the sheet is

26 $$\begin{aligned} \psi (z) = -\frac{\Omega }{2} |z|^2 +\frac{\Gamma }{2\pi }\log |z-z_{pv}|+\frac{\kappa }{2\pi } \log |w| + C+ \sum _{k=1}^N a_{k}\text {Re}(w^{-k}) + b_{k}\text {Im}(w^{-k}), \end{aligned}$$

where the approximation comes from the finite truncation of the series *N*. Here $\kappa$ is the total circulation of the sheet, and the coefficients *C*, $a_k$ and $b_k$ are found using least-squares to satisfy $\psi =0$ on the sheet i.e. the sheet coincides with a streamline, a requirement for equilibrium. Typically in what follows, $N=80$ and the number of sample points on the sheet for applying least-squares is 3*N*.

The first example reproduces, up to some suitable scaling, versions of Figs. 5 and 6 of [9], namely an equilibrium comprising of a point vortex and vortex sheet on the real axis rotating about the origin. This can be thought of as a generalisation of a same-signed point vortex pair in which one of the vortices has been ‘smeared’ out to form a finite length sheet. The total circulation of the sheet is chosen to be equal to that of the point vortex so $\kappa =\Gamma =2\pi$ in (26). Setting $z_{pv}=x_{pv}+i0=1$ implies there are three parameters to be determined: *r*, *c* and $\Omega$. Once found, the circulation density $\rho (x)$ of the sheet (equivalent to the jump in the fluid velocity across the sheet) is calculated using

27 $$\begin{aligned} \rho (x)=-u(x,0^+)+u(x,0^-)=2\text {Im}{{\nabla }\psi }|_{(x,0^+)}, \quad x\in c+r[-1,1], \end{aligned}$$

where the anti-symmetry of $\psi (z)$ about the real axis has been exploited. The derivatives in (27) are computed from (26) using the procedure given in [20].

The parameters $(r,c,\Omega )$ are found by numerical solution using MATLAB’s *fsolve* applied to the three nonlinear equations:

28 $$\begin{aligned} \int _{c-r}^{c+r}\rho (x) \, \textrm{d} x&=\Gamma , \end{aligned}$$

29 $$\begin{aligned} |\nabla \psi |_{z=1}&=0, \end{aligned}$$

30 $$\begin{aligned} \int _{c-r}^{c+r}x\rho (x) \, \textrm{d} x&+x_{pv}\Gamma =0, \end{aligned}$$

where $x_{pv}=1$. Equation (28) states that the circulation of the sheet, $\kappa$, obtained by integrating $\rho (x)$ along the sheet is equal to that of the point vortex $\Gamma$, (29) is the necessary equilibrium condition that the velocity field vanishes at the point vortex, and (30) says that the centre of vorticity, coinciding with the centre of rotation of the equilibria (see e.g. [11]), is the origin. Equation (28) implies that the tips $z_0=c\pm r$ of the sheet coincide with stagnation points, for if they were not, the sheet circulation would be unbounded owing to the $(z-z_0)^{-1/2}$ velocity singularity at the tips.

**Fig. 6:**
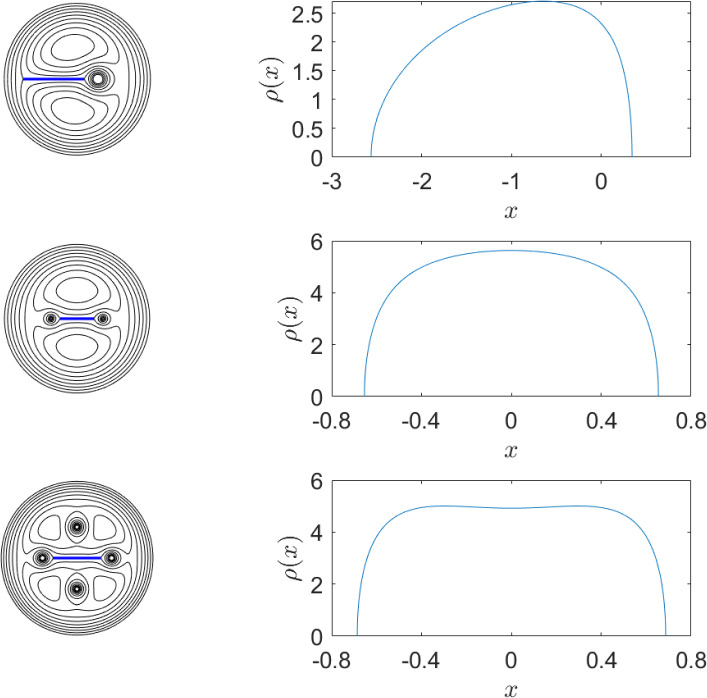
Numerically computed examples of sheet and point vortex equilibria with the left column showing streamlines (black lines), and the sheet (blue line) and the right column showing the corresponding circulation density $\rho (x)$ along the sheet. The circulation of each point vortex in all cases is $\Gamma =2\pi$. Top row: single point vortex at $z=1$; Middle row: two point vortices at $z=\pm 1$; Bottom row: four point vortices at $z=\pm 1$ and $z\approx \pm 0.9005 i$ (colour figure online)

In order to avoid the trivial solution when the sheet collapses to a point vortex of strength $\Gamma$ at $z=-1$ (a point vortex pair) some experimentation with the initial guesses required by *fsolve* is needed. The choice $(r,c,\Omega )_\textrm{guess}=(1,-1,0.5)$ leads to the non-trivial solution $(r,c,\Omega )\approx (1.452,-1.110,0.5889)$ to four significant figures. Other ‘nearby’ initial guesses give the same solution.

A plot of the streamlines and $\rho (x)$ is given in the top row of Fig. 6. The solution can be compared to the exact solution of [9] (see Figs. 5 and 6 of that paper) with some suitable rescaling of the latter to shift the point vortex to $z=1$ and also adjusting the timescale to maintain the vortex circulation of $2\pi$, so giving $\Omega _{exact}=0.5889\cdots$, and, using equations (4) and (11) from [9],

31 $$\begin{aligned} \rho _{1\textrm{vortex}}(x)=2\text {Re}{\left( 4\Omega -\Omega ^2x^2-\frac{1}{(x-1)^2}\right) ^{1/2}}, \end{aligned}$$

from which it can be inferred $(r,c,\Omega )_\textrm{exact}=(1.4524\cdots ,-1.1098\cdots ,0.5889\cdots )$ which agrees with that found using the present numerical least-squares method to 4 significant figures. Moreover, overlaying a plot of the curve given by (31) (not shown) with that in Fig. 6 shows no discernible difference.

Two further examples of equilibria computed using the same numerical procedure are presented, now involving multiple point vortices. Both of these solutions can also be derived exactly using the method of [9] though they do not appear in that work. The first of these consider two vortices at $z=\pm 1$ each having $\Gamma =2\pi$, with a finite vortex sheet aligned with the real axis between the vortices. Again this can be considered a variation of the rotating equilibrium of three identical collinear point vortices in which the middle vortex is smeared out into a finite sheet along the real axis. In this example, the sheet is assumed to have total circulation equal to that of one of the point vortices i.e. $\kappa =\Gamma$. By symmetry, obviously $c=0$ and there are only two unknowns *r* and $\Omega$. The two equations to be solved numerically are (28) and (29). Also by symmetry, the vortex equilibrium condition at $z=-1$ is automatically satisfied if it is at $z=1$, so it suffices to consider (29). Using *fsolve* coupled with the series approximation for the solution of Laplace’s equation and the least-squares method gives $(r,\Omega )\approx (0.6546,1.653)$ to four significant figures. The middle row of Fig. 6 shows the computed streamlines and $\rho (x)$, the latter of which when plotted (not shown) is indistinguishable from the exact solution calculated according to the method of [9]

32 $$\begin{aligned} \rho _{2\textrm{vortex}}(x)=2\text {Re}{\left( 6\Omega -\Omega ^2x^2-\frac{1}{(x-1)^2}-\frac{1}{(x+1)^2}\right) ^{1/2}}. \end{aligned}$$

The next example has four vortices each of circulation $\Gamma =2\pi$, at locations $z=\pm 1$ and $z=\pm i h$ ($h\in \text {Re}$) with a sheet of width 2*r* aligned along the real axis and centred at the origin. In this problem the unknowns are *h*, *r* and $\Omega$ and the equations used are (28) and (29) with the extra equation provided by the requirement that the vortex velocity vanishes at $z=i h$, i.e. $|\nabla \psi |_{z=ih}=0$ (again, by symmetry, it suffices to consider only one of the $z=\pm ih$ vortex locations, along with that at $z=1$). The least-squares numerical method gives to four significant figures $(r,h,\Omega )\approx (0.6866,0.9005,2.793)$, and streamlines and circulation density of the sheet are shown in the bottom row of Fig. 6. Again the method of [9] can be used to derive the exact circulation density giving

33 $$\begin{aligned} \rho _{4\textrm{vortex}}(x)= &   2\text {Re}\bigg (10\Omega -\Omega ^2x^2-\frac{1}{(x-1)^2}-\frac{1}{(x+1)^2} \nonumber \\  &   -\frac{1}{(x-ih)^2}-\frac{1}{(x+ih)^2}-\frac{8\Omega h^2}{x^2+h^2}\bigg )^{1/2}, \end{aligned}$$

which also show no discernible difference when plotted with the numerically generated $\rho (x)$ in Fig. 6.

### Multi sheet equilibria of the Protas-Sakajo class

A family of equilibria involving 2*n* straight vortex sheets are constructed in [10]. Each sheet stems from a common centre with endpoints at the vertices of a regular 2*n*-polygon. The ensemble rotates with angular speed $\Omega$ about its geometric centre. Riemann-Hilbert methods are used in [10] to construct the equilibria, arriving at an integral formula for the circulation density $\rho (x)$ along a given sheet. The integral formula for $\rho (x)$ is evaluated exactly for the case $n=1$, corresponding to the well-known single-sheet equilibria e.g. [7, 8], and also for the case $n=2$ where [10] find a new closed form expression for a four sheet equilibria. For $n>2$ a numerical method for evaluating the singular integral giving $\rho (x)$ is presented [10].

Here, the log-lightning method of [22] is used to find equilibria belonging to the same class. The method accurately solves Laplace’s equation in two-dimensional domains using a series of reciprocal log terms with fitting to boundary data using least-squares. The method is used here in preference to the power series method of 7.1 since it is easily adaptable to handling multiple sheets which are akin to some of the geometries considered in [22].

The angular speed of the structure is fixed at $\Omega =1$, and in the rotating frame the boundary data on the sheets is set by imposing $\psi =\phi - |z|^2/2=0$, and seeking a harmonic function $\phi$ with logarithmic behaviour at infinity and strength equal to the total circulation of the multi-sheet structure. Each sheet has unit length from the origin to its tip with, following [22], 200 computational points exponentially clustered near both ends of a sheet. The total circulation of the vortex sheet structure is found by iteration by ensuring that in the rotating frame the tips of the sheets coincide with stagnation points, this being a necessary condition at equilibrium [10, 30]. Unlike [10], the method is able to compute equilibria for an odd number of sheets.

**Fig. 7:**
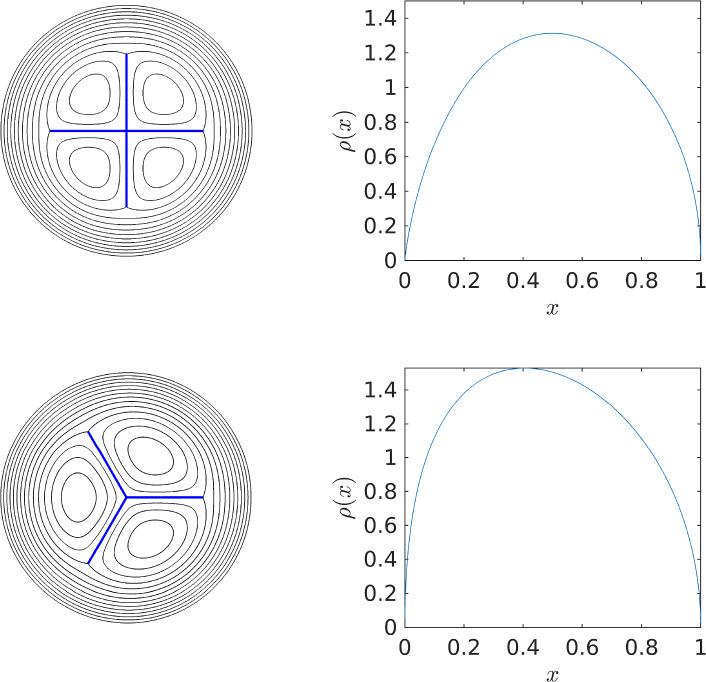
Streamlines (black lines) of the three and four sheet (thick blue lines) equilibrium (left) and the associated circulation density $\rho (x)$ (right) along one of the sheets (colour figure online)

Figure 7 shows streamlines for three and four sheets of unit length and the corresponding circulation density $\rho (x)$ along a sheet. In the four sheet case, comparison can be made to [10]’s exact result, equation (28b)

34 $$\begin{aligned} \rho (x)=\frac{4}{\pi }x\,\textrm{coth}^{-1}\left[ 1/\sqrt{1-x^4}\right] . \end{aligned}$$

Note that (34) differs from [10]’s equation (28b) by a factor $2/\pi$; the factor $\pi$ in the denominator should appear in their result, and the factor two is needed for the choice $\Omega =1$ e.g. [9] and [30].

In the four sheet case, when integrated along one sheet $x\in [0,1]$, the exact result (34) gives when integrated from $x=0$ to $x=1$ a circulation of $\Gamma _{sheet}=1$, so that the total circulation is $4\times \Gamma _{sheet}=4$. This choice is made in the numerical algorithm. Streamlines computed using the log-lightning method are shown in the top row of Fig. 7 along with the numerically determined $\rho (x)$. The latter was obtained by finding the conjugate of the stream function (i.e. the velocity potential) along the sheet aligned with the positive real axis and numerically differentiating this to find the velocity jump across the sheet. A plot of the exact result (34) (not shown) appears identical to that of $\rho (x)$ shown in Fig. 7.

For the three sheet case the total circulation along the sheet is computed iteratively by satisfying the condition at sheet tips i.e. $\rho (1)=0$. This gives $\Gamma _{sheet}\approx 1.214$ to four significant figures. Not unexpectedly, this is greater than the value $\Gamma _{sheet}=1$ for the four sheet equilibria, with the relative increase required in the three sheet equilibria in order for both structures to rotate with $\Omega =1$. Indeed, carrying on this argument for two sheets stemming from the origin (i.e. a single-sheet on $[-1,1]$) also rotating with $\Omega =1$ gives that $\Gamma =\pi$ [30]. Streamlines and circulation density along one of the sheets is shown in the bottom row of Fig. 7. Note that the three sheet case bears resemblance to [11]’s Fig. 6 showing a multiple point vortex approximation to a three sheet equilibrium.

## Steady vortex layers surrounding objects

The two-dimensional free boundary problem in which fluid flows steadily with constant vorticity in a layer $\Omega$ of finite thickness surrounding a solid object *D* has received attention [e.g. 31, 32, 33]. The free boundary is the outer boundary on which the condition that the fluid velocity vanishes is enforced. The identical mathematical problem arises in the dip-coating of objects in which viscous fluid in contact with an object drains under gravity, forming a coating layer surrounding the object [e.g. 34, 35, 36, 37]. These analytical studies have found exact solutions for various objects in the form of infinite wedges or plates of finite length and zero thickness. Finite objects with non-zero area have resisted analysis, and the purpose of this section is to demonstrate that least-squares type methods for solving Poisson’s equation can be used to find accurate numerical solutions for the shape of the free boundary. The additional complexity here is that, for a single finite object, the problem is doubly connected since the vortex layer comprises of two boundaries. One is the curve $\partial D$ representing the object’s boundary on which $\psi$ is constant, the other is the free boundary $\gamma$ on which, without loss of generality, $\psi =\psi _n=0$ which implies the free boundary is a streamline on which the fluid velocity is zero. Fluid outside the vortical layer $\Omega$ is stagnant.

The non-dimensional problem is

35 $$\begin{aligned} \nabla ^2\psi&=1\;\text { in }\Omega , \end{aligned}$$

36 $$\begin{aligned} \psi&=\psi _n=0\;\text { on }\gamma , \end{aligned}$$

37 $$\begin{aligned} \psi&=C\;\text { on }\partial D, \end{aligned}$$

where $\partial D$ is the boundary of the solid in contact with the vortical layer, $\gamma$ is the outer, free boundary and *C* is a constant which controls the layer thickness. The principal task is to determine the free boundary shape $\gamma$.

Following Sect. 3, to solve the Poisson equation (35), let $\psi =\phi +|z|^2/4$, so the problem becomes that of finding a harmonic function $\phi$ with appropriately modified boundary conditions on $\partial D$ and $\gamma$. This is solved by iteration: let the free boundary at the *n*th iteration be $\gamma ^{(n)}$. The harmonic problem for $\phi$ with the Dirichlet boundary conditions $\phi =C-|z|^2/4$ on $\partial D$ and $\phi =-|z|^2/4$ on $\gamma ^{(n)}$ is solved numerically using the doubly-connected version of the AAA-LS algorithm e.g. [23]. The algorithm also computes the gradient of $\nabla \psi$ on $\gamma ^{(n)}$ and this is not necessarily zero (as required by (36)) at the $n^{\text {th}}$ iteration. The radial derivative $\psi _{r_j}=\psi _r|_{r=r_j}$ is found at *N* points $z_j=r_j \exp (i\theta _j)$, $j=1,\cdots , N$, around the boundary $\gamma ^{(n)}$, each point being uniformly spaced in $\theta$ i.e. $\theta _j=(j-1)\Delta \theta$, $\Delta \theta =2\pi /N$.

To satisfy the boundary condition (36), at the $(n+1)^{\text {th}}$ iteration the radial distance $r_k^{(n)}$ is adjusted by an amount $\delta _k$ so that

38 $$\begin{aligned} \psi _{r_j}(r_1^{(n)}+\delta _1,\ldots ,r_k^{(n)}+\delta _k,\ldots ,r_N^{(n)}+\delta _N)=0,\quad j=1,\ldots ,N. \end{aligned}$$

Note that (38) imposes that $\psi _r=0$ on $\gamma$ instead of $\psi _n=0$ as written in (36). In practice, and provided the boundary remains single-valued along a given ray $\theta =$constant, these are equivalent when coupled with the condition $\psi =0$ on $\gamma$ since either choice implies that the velocity vanishes on $\gamma$.

For $|\delta _k|\ll r_k^{(n)}$, $k=1,\cdots ,N$, expanding and retaining first-order terms results in the matrix equation for the perturbations $\delta _k$

39 $$\begin{aligned} \sum _{k=1}^N\delta _k\frac{\partial \psi _{r_j^{(n)}}}{\partial r_k}=-\psi _{r_j}\quad j=1,\ldots ,N. \end{aligned}$$

As in e.g. [12], retaining only the diagonal elements of the matrix and combining with a relaxation parameter $\mu$ is sufficient to obtain good convergence upon iteration. That is,

40 $$\begin{aligned} \delta _k=-\left( \psi _r/\psi _{rr}\right) |_{r=r_k^{(n)}}, \quad k=1,\ldots ,N, \end{aligned}$$

and

41 $$\begin{aligned} r_k^{(n+1)}=r_k^{(n)}+\mu \delta _k, \end{aligned}$$

where the superscript $(n+1)$ indicates the value of $r_k$ at the $(n+1)^{\text {th}}$ iteration. This then gives $\gamma ^{({n+1})}$ using $z_j^{(n+1)}=r_j^{(n+1)} \exp (i\theta _j)$. The choice $\mu =0.8$ is made in the results that follow, with iterations continuing until the root mean square ratio of the variation $\delta _k$ to the radial distance $r_k^{(n)}$ satisfies

42 $$\begin{aligned} \sqrt{\frac{1}{N}\left( \sum _{k=1}^N \delta _k^2\right) }\Bigg /\sqrt{\left( \frac{1}{N}\sum _{k=1}^N {r_k^{(n)}}^2\right) }<10^{-4}. \end{aligned}$$

**Fig. 8:**
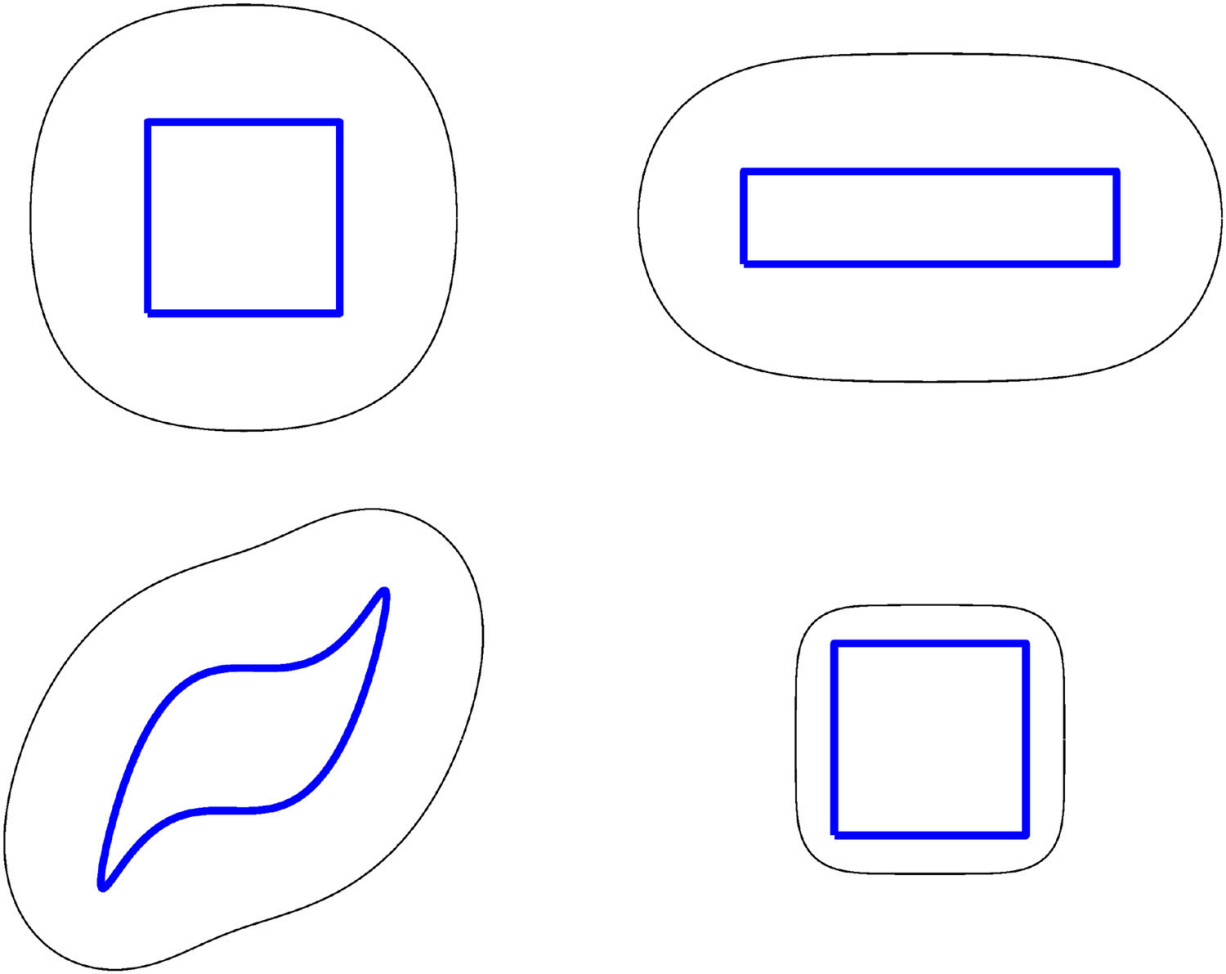
Fluid layers of unit vorticity surrounding objects of unit area taking the form of a square, rectangle (of aspect ratio 4) and a blade shape. The free boundary of the vortex layer is the black line on which $\psi =\psi _n=0$; the blue line is the solid boundary on which $\psi =0.2$, except the bottom right square object which has $\psi =0.02$ (colour figure online)

Figure 8 shows the free boundary for various object shapes each having unit area including a square, a rectangle with aspect ratio 4 and a ‘blade’-shape taken from [38]. For $C=0.2$, then 8, 21 and 8 iterations are required for the square, rectangle and blade, respectively. For the square with $C=0.02$ the vortex layer is thinner and 10 iterations are required.

## Discussion

One objective of this work is to present another application of the two-domain AAA-LS method of Sect. 3, namely its use in vortex dynamics (Sects. 4-6), so adding to its recent application to biological electrostatics [26]. The two-domain AAA-LS method is able to reproduce the velocity field of the Kirchhoff vortex to an $\mathcal {O}(10^{-7})$ degree of accuracy in a matter of seconds on a standard laptop. The two-domain AAA-LS method was then combined with the iterative procedure of [13] to find numerical equilibria of $m-$fold vortex patch scenarios and matched with their results to at least an $\mathcal {O}(10^{-4})$ agreement. A modification to this iterative algorithm as described in [15] then allowed equilibria to the $m+1$ point vortex-vortex patch problem to be found, with example solutions given in Fig. 5

Of course, it should be noted that the standard approach to computing vortex patch equilibria based on contour dynamics [e.g. 13, 14, 15] in which the dynamics is reduced to a line integral around the patch boundary is also an efficient and accurate method. The two-domain AAA-LS method usefully adds to the numerical approaches available to computing vortex patch equilibria.

The examples presented in Sects. 7.1 and 7.2 demonstrate the efficacy of ‘lightning’ numerical methods based on series solution of Laplace’s equation in conjunction with least-squares satisfaction of boundary data for finding vortex sheet equilibria. While not tackled here, the ability of series methods to handle multiple slits (i.e. sheets) [20], including those which are curved and branched [22], shows promise for numerically generating new families of both rotating and translating vortex sheet equilibria. The methods are also suggestive of a new way to find, analytically, vortex sheet equilibria by a combination of conformal mapping of the domain exterior to the sheet to, say, the exterior of the unit disk in an auxiliary plane and and then constructing a Fourier series to satisfy the stream function boundary condition on the sheet. This idea will be taken up in a sequel.

Section 8 demonstrates the AAA-LS method applied to doubly-connected problems by computing the shape of a two-dimensional fluid layer with uniform vorticity surrounding a finite object on which the velocity vanishes on the free boundary. New numerical solutions are presented for a variety of object shapes.

The success of the least-squares methods in this work suggests a new numerical approach for finding new families of vortex equilibria. A combination of the methods discussed here could be used to numerically compute equilibria involving a mix of vortex patches, sheets, points and solid objects. For example, to the authors’ knowledge, there are no known examples of equilibria involving both patches and sheets together. Combining the methods of Sects. 3 and 7 may be one approach to (numerically) find such equilibria. In fact, taking the limit of the layer solution of Sect. 8 where the width of the rectangle vanishes represents a particular class of a sheet-patch solution on which the velocity identically vanishes. Yet the numerical method of Sect. 8 would require further modification since the poles of the interior of the rectangle become too crowded in this limit.

## Data Availability

No datasets were generated or analysed during the current study.

## WOZ numerical algorithm

This appendix summarises the algorithm used by Wu, Overman and Zabusky (WOZ) in [13] to compute $m-$fold symmetric V-states of the vortex patch system (1) − (5). Recall that, by symmetry, only a 1/2*m* segment of the vortex patch boundary is considered which is made up of $M+1$ boundary points. The WOZ numerical routine is split into five steps at each iteration.

First, the velocity on each boundary point must be found, which [13] calculate using contour dynamics techniques, see their equation (4.1). The AAA-LS algorithm is instead used in this work to find the velocity (10) (or (11) as the velocity is continuous across the boundary). Note that the AAA-LS algorithm requires the entire boundary data *Z* rather than just the segment $Z_s$. Furthermore, if the vortex patch has changed shape following an iteration, the new shape must again be non-dimensionalised; it will have a new radius *L* and a new area *A* and thus the non-dimensional constant $\lambda$ must be updated.

Second, the angular velocity is calculated:

A1 $$\begin{aligned} \Omega ^{(n)} = \frac{2}{R_a^2-R_b^2} \sum _{k=1}^M u_{k+1/2}^{(n)}\Delta y_k^{(n)} - v_{k+1/2}^{(n)}\Delta x_k^{(n)}, \end{aligned}$$

where $f_{k+1/2} = \frac{1}{2}[f_{k+1}+f_{k}]$ and $\Delta f_k = f_{k+1}-f_{k}$. Third, the following function is calculated using $\Omega$

A2 $$\begin{aligned} F_{k+1/2} \equiv \frac{u_{k+1/2}\sin {\theta _{k+1}}-v_{k+1/2}\cos {\theta _{k+1}}+(\Omega /2)R_{k+1}}{u_{k+1/2}\sin {\theta _{k}}-v_{k+1/2}\cos {\theta _{k}}+(\Omega /2)R_{k}}. \end{aligned}$$

Fourth, the velocity (*u*, *v*) and angular velocity $\Omega$ are used to evaluate the kinematic boundary condition - the condition that particles on the vortex patch boundary remain on the boundary. The second order discretisation of this condition [13] can be averaged and written as

A3 $$\begin{aligned} -\frac{1}{2}F^{-1}_{k-1/2}\overline{R_{k-1}}+\overline{R_k}-\frac{1}{2}F_{k+1/2}\overline{R_{k+1}} = 0, \;\;\;\; 2\le k\le M, \end{aligned}$$

where $\overline{R_1}=R_a$, $\overline{R}_{M+1}=R_b$ and *F* is the function given in (A2). The system (A3) can be written as the product $AX=Y$, where *A* is a tridiagonal matrix of coefficients and *Y* is the vector $(\frac{1}{2}F^{-1}_{3/2} R_a, 0, 0, ... 0, \frac{1}{2}F_{M+1/2} R_b)$. The vector of unknowns $X=(\overline{R_2},...,\overline{R_M})$ can then be found using the MATLAB backslash operator $X = A\backslash Y$ and it follows that $\overline{R_k}\rightarrow \overline{R_k}^{(n+1)}$. The following relaxation procedure is then used

A4 $$\begin{aligned} R_k^{(n+1)}=\mu \overline{R_k}^{(n+1)}+(1-\mu )R_k^{(n)}, \end{aligned}$$

where $\mu =0.6$ as in [13].

Fifth, the boundary data *Z* and segment data $Z_s$ are updated accordingly. Then, the iteration continues until the following error criterion is satisfied

A5 $$\begin{aligned} \sum _{k=1}^{M+1}|R_k^{(n+1)}-R_k^{(n)}|<\epsilon , \end{aligned}$$

for some tolerance $\epsilon =\mathcal {O}(10^{-6})$ or until some maximum number of iterations $n_{max}$ is achieved; such an $n_{max}$ is implemented to avoid exceptionally long runtimes. In this work, the value $n_{max}=500$ was used, yet all results presented in this work converged to an equilibrium in fewer iterations.
